# Correlation between Colon Perfusion and Postoperative Fecal Output through a Transanal Drainage Tube during Laparoscopic Low Anterior Resection

**DOI:** 10.3390/cancers14092328

**Published:** 2022-05-08

**Authors:** Kenji Kawada, Toshiaki Wada, Takehito Yamamoto, Yoshiro Itatani, Koya Hida, Kazutaka Obama

**Affiliations:** 1 Department of Surgery, Graduate School of Medicine, Kyoto University, Kyoto 606-8507, Japan; wt0520@kuhp.kyoto-u.ac.jp (T.W.); takehito_y@kuhp.kyoto-u.ac.jp (T.Y.); itatani@kuhp.kyoto-u.ac.jp (Y.I.); hidakoya@kuhp.kyoto-u.ac.jp (K.H.); kobama@kuhp.kyoto-u.ac.jp (K.O.); 2 Department of Surgery, Faculty of Medicine, Kindai University, Osaka 589-8511, Japan; 3 Department of Gastroenterological Surgery and Oncology, Kitano Hospital Medical Research Institute, Osaka 530-8480, Japan

**Keywords:** rectal cancer, ICG angiography, transanal drainage tube, anastomotic leakage

## Abstract

**Simple Summary:**

Anastomotic leakage (AL) is a major problem in patients who undergo operations for rectal cancers. Various solutions, such as ICG angiography and transanal drainage tubes (TDT), have been proposed to prevent AL. Although the microbiota has recently been recognized to play a role in the pathogenesis of AL, mainly from the results of experimental animal models, it remains unclear whether this mechanism can occur in humans. In this study, we investigated the relationship between intestinal perfusion and fecal volume through TDT in laparoscopic low anterior resection, and found that the need of a proximal shift of the transection site due to insufficient intestinal perfusion was significantly associated with high fecal volume, which might reflect the correlation between intestinal perfusion and postoperative diarrhea. In addition, we found that the intensity of ICG fluorescence at the transection site was significantly associated with fecal volume through TDT. To the best of our knowledge, this is the first clinical study to examine the relationship between intestinal perfusion and fecal volume through TDT.

**Abstract:**

In order to prevent anastomotic leakage (AL) following rectal surgery, various solutions—such as intraoperative indocyanine green (ICG) angiography and transanal drainage tubes (TDT)—have been proposed. This study investigated the relationship between intestinal perfusion and fecal volume through TDT in laparoscopic low anterior resection (LAR). A total of 59 rectal cancer patients who underwent laparoscopic LAR with both intraoperative ICG angiography and postoperative TDT placement were retrospectively analyzed. The relationship between intestinal perfusion and fecal volume through TDT was examined. Based on the ICG fluorescence, the transection site was shifted more proximally in 20 cases (33.9%). Symptomatic AL occurred in seven patients (11.8%). The AL rate of the patients whose daily fecal volume exceeded 100 mL/day in 2 or more days was significantly higher than that of those whose daily fecal volume exceeded it in 0 or 1 day (44.4% vs. 6.0%; *p* < 0.01). Univariate and multivariate analyses showed that the need for a proximal shift of the transection site was significantly associated with a high fecal volume. The quantitative analysis of ICG fluorescence indicated that Fmax (the fluorescence difference between the baseline and maximum) was significantly associated with fecal volume through TDT.

## 1. Introduction

Recent advances in minimally invasive surgery, such as laparoscopic surgery and robotic surgery, as well as in stapling devices, have made it possible to dissect and anastomose the rectum close to the anus in rectal cancer surgery. Despite advanced improvements in surgical procedures and perioperative management, anastomotic leakage (AL) following the low anterior resection (LAR) of the rectum is reported to still occur in 3% to 20% of cases; the most commonly reported rates are approximately 10–13% according to the recent large population databases in the USA and Japan [1,2,3]. AL is one of the most destructive complications following rectal surgery, and can result in not only short-term outcomes (morbidity, mortality, and increased length of hospital stay) but also long-term outcomes (overall survival, disease-free survival and local recurrence) [4,5,6]. Although several factors—including BMI, sex, level of anastomosis, tumor size, intestinal perfusion, precompression before stapler firings, diverting stoma, the transanal tube and intestinal microbes—have been reported as possible causes of AL [7,8,9,10,11], the pathogenesis of AL is still unclear. Various attempts, such as intraoperative indocyanine green (ICG) angiography and transanal drainage tube (TDT), have been proposed to prevent AL. Intraoperative ICG angiography is a promising technology to evaluate the intestinal perfusion in real-time [12,13]. We previously reported that the quantitative analysis of ICG fluorescence was beneficial in order to determine the appropriate transection line of the proximal colon, which could reduce the AL [14,15,16]. In recent years, the effectiveness of TDT in the prevention of AL has been widely accepted [17,18,19]. In theory, TDT could be useful in decreasing the endoluminal pressure at the anastomosed colon. We previously reported that the AL rate of the patients whose daily fecal volume through TDT exceeded 100 mL/day in 2 or more days was significantly higher than that of those who exceeded in 0 or 1 day [20].

The intestinal microbiota plays a key role in the pathogenesis of obesity, inflammatory bowel disease, and gastrointestinal malignancies. Although several factors affect AL development, the microbiota has been recently recognized to play a role in the pathogenesis of AL [21,22,23]. The genus *Serratia* was reported to be associated with postoperative AL by producing collagenase in a mouse model [24]. Using experimental rat models, Shogan et al. recently reported that intestinal devascularization altered *Enterococcus feacalis* to become more virulent (i.e., by degrading collagen and activating matrix metalloproteinase), contributing to AL [25]. However, it remains unclear whether intestinal perfusion can affect the microbiota at the anastomotic site in humans. In this study, the need for a proximal shift of the transection site based on ICG fluorescence showed a statistical significance for association with fecal discharge through TDT. Moreover, the quantitative analysis of ICG fluorescence showed that Fmax (the fluorescence difference between the baseline and maximum) was significantly associated with fecal discharge through TDT. As far as we know, this is the first clinical study to examine the relationship between intestinal perfusion and fecal discharge through TDT.

## 2. Materials and Methods

A total of 59 rectal cancer patients who underwent laparoscopic LAR and double-stapling technique (DST) anastomosis with both intraoperative ICG angiography and postoperative TDT placement between October 2013 and January 2018 in our institution were analyzed. Patients with a covering stoma were excluded. All of the patients received mechanical preparation (12 mg pursennid and 75 mg sodium picosulfate) and antibiotic prophylaxis (750 mg metronidazole and 1 g kanamycin) preoperatively. The laparoscopic surgical procedures were performed by board-certified laparoscopic surgeons [26,27]. After the mobilization of the left-sided colon, tumor-specific mesorectal excision—including total mesorectal excision (according to the tumor location)—was performed by the standard surgical technique. The main principle of this technique is sharp mesorectal dissection with a nerve-preserving technique. After clamping distal to the tumor for washout, the rectum was transected using the linear stapler. After the extraction of the specimen through the small incision, the colonic mesentery and marginal vessels were divided at a point which was appropriate for the proximal margin. Subsequently, ICG (5 mg) was injected intravenously, and the blood flow was examined using an NIR camera system (PDE-neo System; Hamamatsu Photonics K.K., Hamamatsu, Japan), as described previously [14]. When the ICG fluorescence of the proposed transection site was judged to be sufficient, the proximal colon was transected as initially planned. When the ICG fluorescence of the initially planned transection site was judged to be insufficient, the transection site was shifted to a more proximal site which was considered to have appropriate ICG fluorescence. The circular stapler was inserted through the rectum, and then end-to-end DST anastomosis was completed intracorporeally. The height of anastomosis from the anal verge was measured by digital examination during anesthesia. Cases converted to a transanal hand-sewn coloanal anastomosis were excluded. After the completion of the DST anastomosis intracorporeally, a Malecot catheter (28 Fr) was placed 5–6 cm above the anastomotic portion. A Malecot catheter was connected to a bag, and the volume of fecal discharge was measured daily, as described previously [25]. The timing of the TDT removal was determined at the surgeon’s discretion. A soft diet was resumed on postoperative days (PODs) 4 or 5, unless obstructive symptoms occurred. 

**Figure 1:**
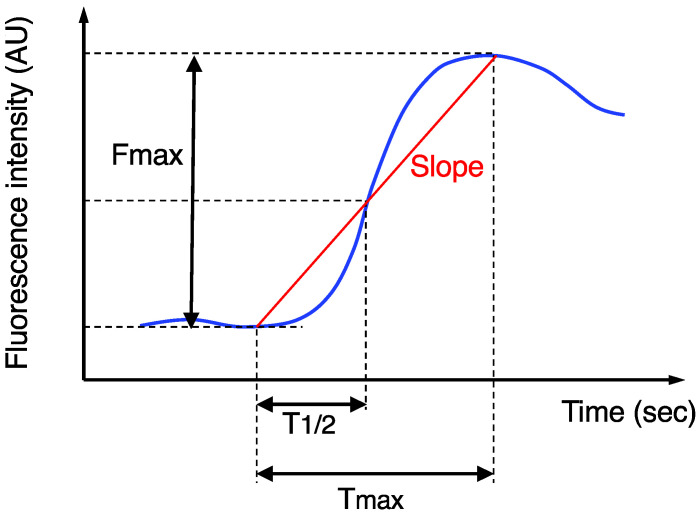
Time curve of the ICG fluorescence intensity. Fmax, Tmax, T1/2, and Slope were calculated.

The imaging data after ICG injection were continuously recorded, and then the stored video files were retrospectively analyzed using a piece of software, ROIs (Hamamatsu Photonics K.K., Hamamatasu, Japan), for the quantitative analysis of the ICG fluorescence, as previously described [14]. The region-of-interest was set at the actual transection portion of the proximal colon, and then the following four parameters were measured (Figure 1): Fmax (fluorescence difference between the baseline and maximum), Tmax (time from ICG onset to the maximum intensity), T1/2 (time from ICG onset to half of the maximum) and Slope (Fmax/Tmax). AL was confirmed by digital examination and radiographic examination (e.g., the extravasation of water-soluble contrast enema, abscess at the level of anastomosis, and fluid/air bubbles surrounding the anastomosis), as previously described [9,14,15,16,20]. Based on the grading system [28], AL was classified into three grades: grade A did not need any intervention; grade B required active intervention; and grade C required reoperation. Only symptomatic AL (grades B and C) was analyzed in this study, because routine contrast enemas to detect asymptomatic AL (grade A) were not performed in our institution. The diagnosis of AL was made within 30 days after the surgery.

Fisher’s exact test was used for the comparison and analysis of the categorical variables. The continuous variables were determined by the Mann-Whitney *U* test. All of the analyses were two-sided, and a *p* value of <0.05 was considered statistically significant. In order to determine factors associated with high fecal volume, multivariate logistic regression analysis was used, and factors with a *p* value of <0.05 were included in the model. The statistical analyses were conducted with the JMP Pro software, version 11.0.0 (SAS Institute Inc., Cary, NC, USA).

The study protocol was approved by the institutional review board of Kyoto University Hospital (R0882) and conformed to the Declaration of Helsinki. Informed consent was obtained from all of the participants.

## 3. Results

Fifty-nine patients (39 male and 20 female) who underwent elective laparoscopic LAR and DST anastomosis with the intraoperative use of ICG angiography were analyzed. The tumor was located within 10 cm of the anal verge in all cases. Forty-six patients (78%) had upper rectal cancer, and the remaining 13 patients (22%) had lower rectal cancer. The median body mass index (BMI) was 22.2 kg/m^2^ (range, 17.1–32.6 kg/m^2^). The high ligation of the inferior mesenteric artery (IMA) was performed in 57 cases (97%), whereas low ligation was performed in two cases (3%). Preoperative chemotherapy was carried out in six patients (14.4%), whereas patients who received preoperative chemoradiotherapy were not included because of the construction of a covering stoma. Lateral lymph node dissection was performed in three cases (5.0%). No adverse events related to ICG angiography and/or TDT placement were observed. No fatal event was observed.

After the assessment of the intestinal perfusion of the proposed transection site under normal white light, ICG was intravenously injected for the evaluation of the intestinal perfusion in the fluorescent mode. Based on the ICG fluorescence, the surgeons opted for a further proximal shift of the transection site to an adequate fluorescence portion in 20 cases (33.9%). The median proximal change distance was 2.0 cm (range, 1.0–6.5 cm).

Symptomatic AL (grades B and C) occurred in seven patients (7/59; 11.8%). In most cases, the TDT was removed between PODs 4 and 7 (range, 1–11). If there was persistent massive discharge, the tube was retained for a longer duration. Based on the postoperative fecal volume through TDT, the patients were classified into two groups: patients whose daily fecal volume exceeded 100 mL/day in 0 or 1 day (the low TDT discharge group; *n* = 50 (84.7%)) and patients who exceeded in 2 or more days (the high TDT discharge group; *n* = 9 (15.3%)). The AL rate of the high TDT discharge group was significantly higher than that of the low TDT discharge group (44.4% (4/9) vs. 6.0% (3/50); *p* = 0.007). These results indicated that the postoperative fecal volume through TDT could be a reliable predictor of AL. We performed a test for *Clostridium difficile* toxin in cases with a high fecal volume, and found that all of the cases were negative.

We next investigated the risk factors for a high fecal volume through TDT (i.e., ≥100 mL/day in 2 or more days) in this series (Table 1). Univariate analysis showed that high fecal volume was significantly associated with a longer operation time (≥300 min), male sex, steroid therapy, and the proximal change of the transection site. Meanwhile, no significant difference was observed in age, serum hemoglobin and albumin levels, BMI, anticoagulation therapy, diabetes mellitus, smoking, preoperative chemotherapy, tumor size, location, intraoperative blood loss, level of IMA ligation, and lateral lymph node dissection. Multivariate analysis including factors with a *p* value of ≤0.05 indicated that only the proximal change of the transection site remained significantly associated with a high fecal volume (*p* = 0.004; odds ratio, 43.6; 95% confidence interval, 3.43–554.2).

**Figure 2:**
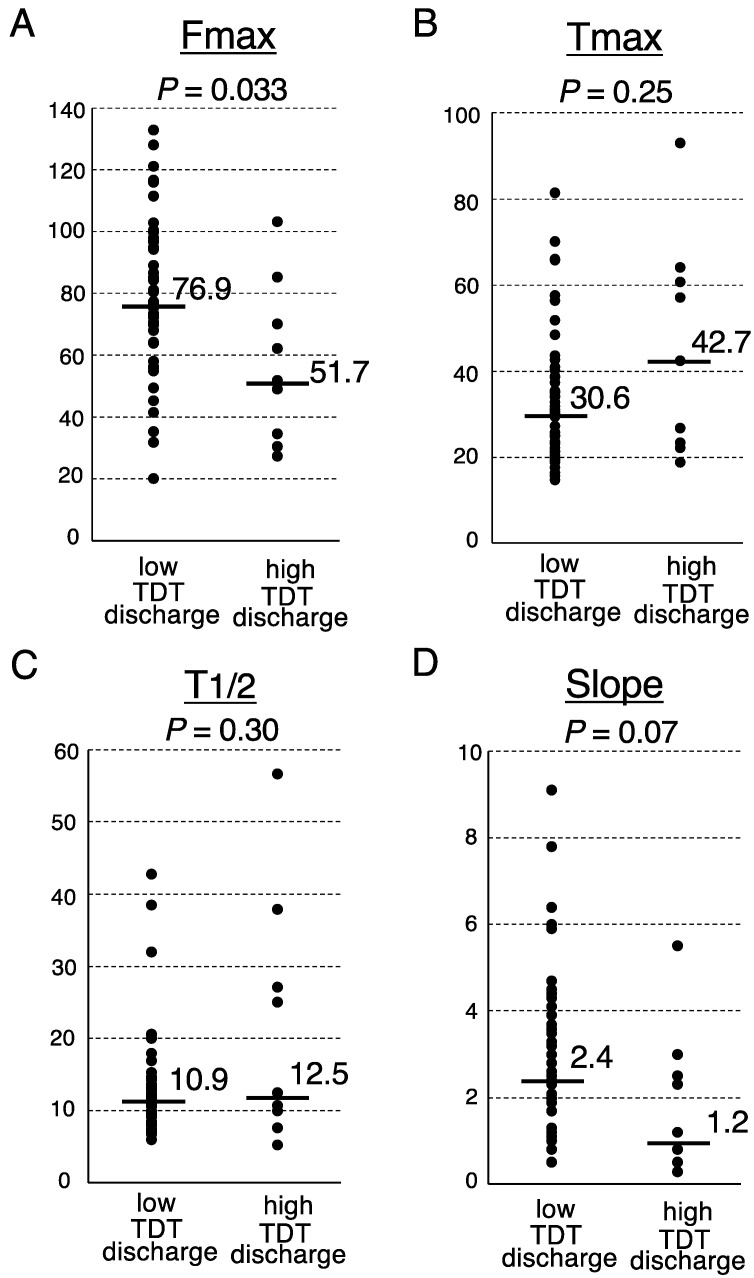
Analysis of the ICG fluorescence-related parameters according to the TDT discharge volume. Fmax (**A**), Tmax (**B**), T1/2 (**C**) and Slope (**D**). Medians, bars. Mann-Whitney *U* test.

Finally, we investigated the association between the postoperative fecal volume and ICG fluorescence-related parameters at the transection portion of the proximal colon (Figure 1). The Fmax of the low TDT discharge group was significantly higher than that of the high TDT discharge group (median, 76.9 arbitrary units (AU) vs. 51.7 AU, respectively; *p* = 0.033, Mann-Whitney *U* test) (Figure 2). The Slope of the low TDT discharge group tended to be higher than that of the high TDT discharge group (median 2.4 vs. 1.2 AU/sec, respectively; *p* = 0.07). Meanwhile, no significant difference was observed between the two groups regarding the Tmax or T1/2.

The clinical characteristics of seven symptomatic AL cases are shown in Table 2. Symptomatic AL needing reoperation (grade C) occurred in 10.2% of the patients (6 of 59), 33.3% of the high TDT discharge group (3 of 9), and 6.0% of the low TDT discharge group (3 of 50). In six patients of the AL cases, the surgical team changed the initially planned transection site of the proximal colon due to the “hypoperfused” appearance on the ICG fluorescence. Symptomatic AL occurred in six (6/20: 30.0%) of the patients who had a revision of the transection site based on ICG angiography, while it occurred in one case (1/39: 2.6%) of the patients who did not need a revision.

## 4. Discussion

An adequate blood supply is a prerequisite for the proper healing of the anastomosis. ICG angiography is a feasible procedure, and it offers promising results in reducing AL [12,13]. When vascular perfusion is assessed to be poor based on ICG fluorescence, the transection site needs to be shifted to the proximal site where the perfusion is considered to be good. In the present study, the transection site was shifted more proximally in 33.9% (20/59). The high ligation of IMA was performed in almost all cases (57/59), which may also affect the results of this study (33.9%). In the previous literature, this figure was highly variable, and ranged from 1.6% to 40% [29]. This could be partially explained by the variation in the operation procedure and methodology used; the present study focused on laparoscopic LAR, and utilized an extracorporeal fluorescence system. In addition, the assessment of the ICG fluorescence intensity was subjective, and—from the surgeon’s visual interpretation of ICG fluorescence—it is sometimes difficult to judge whether the intestinal perfusion is enough or not. This study demonstrated that the proximal change of the transection site was strongly associated with high fecal discharge through TDT (Table 1), which might reflect the correlation relationship between intestinal perfusion and postoperative diarrhea.

The quantitative analysis of ICG fluorescence has been introduced to overcome the subjective interpretation and observer bias of the ICG fluorescence imaging [30,31]. However, no consensus exists regarding the optimal quantitative parameters or the adequate threshold. We previously reported that the quantitative analysis of ICG fluorescence was beneficial for assessing anastomotic perfusion in laparoscopic rectal surgery, and that Fmax was the most predictive for AL among several parameters [14]. We examined the utility of quantitative analysis in this series, and found that the Fmax at the transection site was significantly higher in the low TDT discharge group than in the high TDT discharge group (Figure 2). Considering the results of the recent animal study showing that *Enterococcus feacalis* becomes more virulent by intestinal ischemia [25], we speculate that the hypo-perfused microenvironment in the proximal colon might make the intestinal bacteria more virulent, which could result in increased daily fecal volume and/or AL. When patients require a revision of the transection site based on the ICG angiography, surgeons might pay attention to the fecal discharge through TDT and extend the timing of the TDT removal for the increased fecal volume.

Regarding the fecal discharge through TDT, we previously reported that daily fecal volume gradually increased until PODs 3–4, and then significantly decreased on POD 5, and that the AL rate of the patients whose daily fecal volume exceeded 100 mL/day in 2 or more days was significantly higher than that of those who exceeded in 0 or 1 day (26.9% vs. 7.9%; *p* < 0.01) [20], which was in agreement with the result of this study. In addition, Hidaka et al. also reported that the total volume of fecal discharge for PODs 1–3 after laparoscopic LAR was significantly associated with the rate of AL [32]. Patients undergoing colectomy have a risk of *Clostridium difficile* infection because of the disruption of the colonic microflora. The impact of *Clostridium difficile* infection was reported to increase the number of overall worse outcomes following colectomy [33]. It was reported that postoperative diarrhea or high stoma output regardless of *Clostridium difficile* infection could significantly increase the number of surgical site infections, including AL [34], which may suggest the relationship between AL and the intraluminal pressure increase from postoperative diarrhea. Further investigation focusing on intestinal microbes is required in order to investigate the causes of AL.

Our study has limitations. First, selection bias may have been introduced by selecting patients who underwent laparoscopic LAR with intraoperative ICG angiography and postoperative TDT placement. However, this is inevitable because our study was intended to investigate the relationship between intestinal perfusion and fecal discharge through TDT. Second, the sample size was small. A study in a large number of patients is needed in order to clarify the association between intestinal perfusion and fecal discharge through TDT. We suppose that the next step is to analyze the metabolome and gut microbiome in patients with high fecal discharge through TDT.

## 5. Conclusions

In this study, we report that the need for the proximal shift of the transection site was significantly associated with high fecal volume through TDT, and that quantitative ICG fluorescence intensity (i.e., Fmax) at the transection portion of the proximal colon was significantly associated with fecal volume through TDT. This is the first clinical study to examine the relationship between intestinal perfusion and fecal volume through TDT.

## Figures and Tables

**Table 1:** Univariate and multivariate analyses of the risk factors associated with a high fecal volume.

Variables	Patients with High TDT Discharge n%	Univariate Analysis *p*-Value	Multivariate Analysis *p*-Value OR
Age, years		0.97	
<70	6/39 15.4		
≥70	3/20 15.0		
Sex		0.020	0.99
Male	9/39 23.1		
Female	0/20 0.0		
BMI, kg/m^2^		0.22	
<25	6/48 12.5		
≥25	3/11 27.3		
Albumin, g/dL		0.98	
<4.0	4/26 15.4		
≥4.0	5/33 15.2		
Hemoglobin, g/dL		0.06	
<11	3/8 37.5		
≥11	6/51 11.8		
Location		0.99	
Upper	7/46 15.2		
Lower	2/13 15.4		
Operation time, min		0.039	0.056
<300	1/25 4.0		
≥300	8/34 23.5		
Blood loss, mL		0.53	
<50	8/48 16.7		
≥50	1/11 9.1		
Tumor size, cm		0.80	
<5.0	5/35 14.3		
≥5.0	4/24 16.7		
T category		0.42	
Tis, T1, T2	2/20 10.0		
T3, T4	7/39 17.9		
N category		0.11	
N0	3/34 8.8		
N1, N2	6/25 24.0		
Ligation of IMA		0.16	
high ligation	8/57 14.0		
low ligation	1/2 50.0		
LLND		0.45	
Yes	0/3 0.0		
No	9/56 16.1		
Preoperative chemotherapy		0.92	
No	1/6 16.7		
Yes	8/53 15.1		
Anastomosis level from AV, mm		0.30	
<40	3/28 10.7		
≥40	6/31 19.4		
Diabetes Mellitus		0.19	
No	7/53 13.2		
Yes	2/6 33.3		
Anticoagulation Therapy		0.30	
No	7/52 13.5		
Yes	2/7 28.6		
Steroid therapy		0.045	0.09
No	7/55 12.7		
Yes	2/4 50.0		
Smoking, Brinkman index		0.63	
<400	5/37 13.5		
≥400	4/22 18.2		
Proximal change of resection site		0.003	0.004 43.6
No	2/39 5.1		
Yes	7/20 35.0		

Abbreviations: LLND, lateral lymph node dissection; OR, odds ratio.

**Table 2:** Clinical characteristics of the AL cases.

Case	AL Grade	Sex	Operation Time (Min)	Diabetes Mellitus	Anticoagulation Therapy	Steroid Therapy	Fecal Discharge through TDT	Change of Resection Site	Fmax at Transection Site	Slope at Transection Site
1	C	male	≥300	No	No	No	high	Yes	27.2	0.3
2	C	male	≥300	Yes	No	No	high	Yes	30.6	0.5
3	C	male	≥300	Yes	No	No	low	No	49.4	2.0
4	C	male	≥300	No	No	No	low	Yes	31.8	1.3
5	B	male	≥300	No	Yes	No	high	Yes	51.7	0.8
6	C	male	<300	No	No	No	high	Yes	49.1	1.2
7	C	female	<300	No	No	No	low	Yes	20.2	0.5

## Data Availability

No new data were created or analyzed in this study. Data sharing is not applicable to this study.
